# Molecular mechanism of UV damage modulation in nucleosomes

**DOI:** 10.1016/j.csbj.2022.08.071

**Published:** 2022-09-14

**Authors:** Bastian Stark, Gregory M.K. Poon, John J. Wyrick

**Affiliations:** a School of Molecular Biosciences, Washington State University, Pullman, WA 99164, USA; b Department of Chemistry and Center for Diagnostics and Therapeutics, Georgia State University, Atlanta, GA 30303, USA; c Center for Reproductive Biology, Washington State University, Pullman, WA 99164, USA

**Keywords:** UV damage, Cyclobutane pyrimidine dimers, CPD, Histones, Photofootprint, Mutations

## Abstract

Exposure to ultraviolet (UV) light causes the formation of mutagenic cyclobutane pyrimidine dimers (CPDs) in cellular DNA. Previous studies have revealed that CPD formation in nucleosomes, the building blocks of chromatin, shows a striking ∼10 base pair (bp) periodic pattern. CPD formation is suppressed at positions where the DNA minor groove faces toward the histone octamer (minor-in) and elevated CPD formation at positions where the minor groove faces away from the histone octamer (minor-out). However, the molecular mechanism underlying this nucleosome photofootprint is unclear. Here, we analyzed ∼180 high-resolution nucleosome structures to characterize whether differences in DNA mobility or conformation are responsible for the CPD modulation in nucleosomes. Our results indicate that differences in DNA mobility cannot explain CPD modulation in nucleosome. Instead, we find that the sharp DNA bending around the histone octamer results in DNA conformations with structural parameters more susceptible to UV damage formation at minor-out positions and more resistant to CPD formation at minor-in positions. This analysis reveals the molecular mechanism responsible for periodic modulation of CPD formation and UV mutagenesis in nucleosomal DNA.

## Graphical abstract

**Figure:**
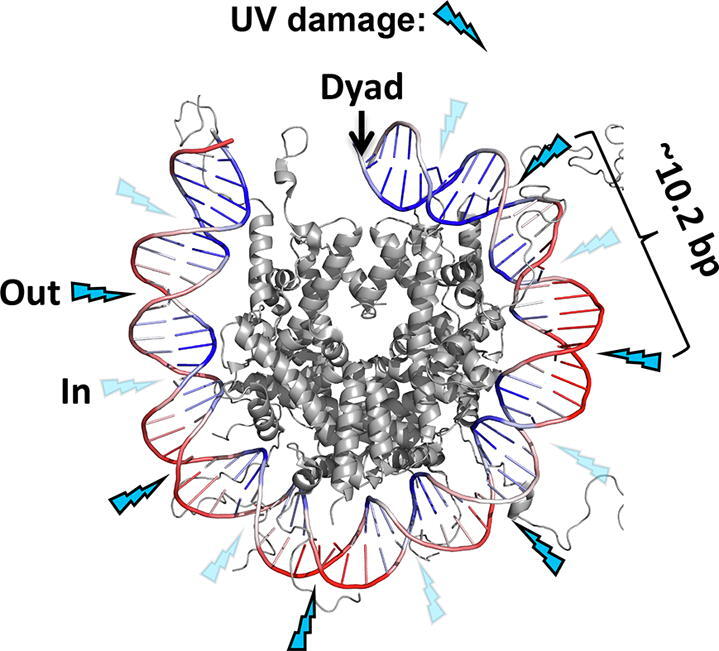

## 1 Introduction

Ultraviolet (UV) light is the primary etiologic agent for skin cancers such as melanoma because it induces mutagenic lesions in DNA. The primary DNA lesion induced by UV is the cyclobutane pyrimidine dimer (CPD), which comprises approximately 80 % of UV damage to DNA [1]. These helix-distorting DNA lesions are significant obstacles to both RNA and DNA polymerases, and error-prone bypass of CPD lesions during replication is thought to be the primary cause of UV-induced mutations in skin cancers [1], [2], [3]. CPDs form via a rapid [2+2] cycloaddition reaction between the C5-C6 double bonds of neighboring pyrimidine bases, resulting in covalent cross-links between the adjacent pyrimidines [4]. In canonical B-form DNA, however, the quantum yield of this reaction is very low [4], [5], [6], [7]. This is likely because the distance between and alignment of the neighboring C5-C6 double bonds in canonical B-form DNA is unfavorable to the 2+2 cycloaddition reaction [4], [5], [6], [7]. It is thought that CPD formation may occur in part through fluctuations in the canonical DNA structure, resulting in transient conformations with more favorable distance and alignment parameters that more readily form CPDs upon UV absorbance [4], [5], [6], [7]. An implication of this model is that alterations in DNA conformation or mobility due to DNA-bound proteins could potentially alter the rate of UV-induced CPD formation.

CPD formation in human cells is significantly modulated by the packaging of DNA into chromatin [8], [9], [10], [11], [12], [13]. The primary building block of chromatin is the nucleosome, comprised of ∼147 bp of DNA wrapped nearly two times around an octamer of histone proteins [14]. The nucleosomal DNA is strongly bent as it wraps around the histone proteins, particularly at positions every 10 bp where the DNA minor groove faces the histone octamer [14], [15], [16], [17]. The histones directly contact the DNA sugar-phosphate backbone at these ‘minor-in’ rotational settings, resulting in constrained DNA mobility and sharp bending into the minor groove through alterations in the roll and slide parameters of the DNA base stack [14], [15]. Previous biochemical studies of damage formation in UV-irradiated cells or isolated nucleosomes indicates that CPD formation is suppressed at minor-in positions [8], [12]. In contrast, CPD formation is elevated at ‘minor-out’ positions, where the DNA minor groove faces away from the histone octamer. This results in a periodic pattern of CPD formation in nucleosomes, with peaks every ∼10 bp at minor-out positions, which is collectively known as the nucleosome photofootprint [8], [12]. More recent studies using genome-wide approaches to map CPD formation at single nucleotide resolution in both yeast and human cells have confirmed that nucleosomes cause this periodic pattern of CPD formation across the genome, especially when nucleosomes are strongly positioned [9], [10], [11], [13]. Importantly, this periodic trend of CPD formation in human nucleosomes is mirrored by a similar pattern of mutations in human skin cancers, in which somatic mutations are elevated at minor-out positions and suppressed at minor-in rotational settings [9], [10], [11], [12], [13], [18].

While these studies have established that the rotational setting of nucleosomal DNA impacts both CPD formation and mutation rates in skin cancers, the molecular mechanism responsible for this photofootprint is unclear. It was originally suggested that decreased DNA mobility at minor-in positions, presumably due to constraints imposed by direct histone contacts, and elevated DNA mobility at minor-out positions might be responsible for the pattern of CPD formation in nucleosomes [8], [12]. Alternatively, it has been suggested that sharp bending of the DNA into the major or minor groove as it wraps around the histone octamer could cause essentially static DNA conformations that were more or less susceptible to CPD formation [19], [20], [21]. However, since these models were proposed prior to the publication of high resolution structures of the nucleosome, they have not been rigorously tested. Moreover, the exact nature of the DNA conformation adopted at minor-in and minor-out positions and the mechanism by which it modulates CPD formation is unknown.

We and others have recently shown that other DNA-bound proteins, including the transcription factors CTCF and members of the E26 transformation-specific (ETS) family, also significantly modulate CPD formation at their DNA binding sites in human cells [22], [23], [24], [25], [26]. Analysis of high-resolution structures of these transcription factors bound to DNA revealed a common molecular mechanism responsible for the change in UV damage formation at their binding sites. For both ETS and CTCF, binding-associated changes in the distance and relative torsion angle of the C5-C6 atoms of neighboring pyrimidines could in many cases explain the observed CPD induction [22], [23], [27], [28]. For example, both ETS and CTCF binding decreased the distance and torsion angle to more favorable values at sites of CPD hotspots in the binding sites. These findings are consistent with biophysical studies indicating that these structural parameters may regulate the frequency of CPD formation [4], [6], [7]. However, whether this mechanism could potentially explain the modulation of CPD formation at nucleosomes is unclear.

The elucidation of numerous high-resolution structures of nucleosomes containing diverse DNA sequences provides a unique opportunity to revisit the molecular mechanism responsible for the nucleosome photofootprint. Here, we analyzed ∼180 high-resolution structures of nucleosomes to answer this question.

## 2 Experimental Procedures

### 2.1 CPD-seq data analysis

We analyzed published CPD-seq data from UV-irradiated yeast cells or yeast naked DNA [9], as described previously. CPD lesions were assigned to either half-integer positions to represent the two pyrimidine positions comprising the CPD [23], or at single integer positions (i.e., a lesion was assigned to both bases that comprise the CPD). The locations of ∼10,000 strongly positioned nucleosomes in yeast were obtained from [29], and CPD-seq data in these strongly positioned nucleosomes were analyzed as described previously [9], [30]. CPD enrichment was determined by normalizing cellular CPD-seq reads (UV 0hr) to the naked DNA control at each position in the nucleosomal DNA (i.e., positions −73.5 to +73.5 or −73 to +73) relative to the nucleosome dyad. For most of the analysis, CPD-seq reads were combined for symmetric positions across the dyad (e.g., weighted average of position −10 and +10, etc.).

### 2.2 Compendium of nucleosome structures

We identified and obtained atomic coordinates of nucleosome structures from the PDB. Only high-resolution structures with a resolution of no greater than 3.50 Å were included in the final analysis. 181 distinct structures fit these criteria (Supplementary Table S1). We also identified the base pair corresponding to the central dyad axis of each DNA chain in the nucleosome structure. For analysis of B-factor in linker DNA regions, we analyzed a relatively high resolution tetranucleosome structure (PDB ID: 5OY7).

### 2.3 DNA mobility analysis

B-factor was used to quantify DNA mobility. A custom python program was used to retrieve B-factor values from the compendium of 181 nucleosome structures for each atom within the DNA backbone (P, OP1, OP2, O5′, C5′, C4′, O4′, C3′, O3′, *C*2′, C1′) and assigned to positions corresponding to individual nucleotides. These were averaged for each position to generate a single B-factor value associated with each position in the nucleosomal DNA. B-factor values were calculated for nucleosomal DNA positions −73 to +73 relative to the nucleosome dyad axis and combined for symmetric positions across the nucleosome dyad (i.e., position −10 and +10 were combined and averaged; see above). Comparison with CPD enrichment was performed using Pearson correlation analysis in Graphpad Prism software. B-factors were also normalized (B_Norm_; see [31]) using a z-score derived from the average and standard deviation of B-factors averaged for each DNA residue in a structure.

For the B-factor analysis of the tetranucleosome structure, we analyzed B-factor for the normal antiparallel orientation of the DNA strands and for strand-aligned (i.e., both DNA strands in the 5′-3′ orientation with linker regions aligned) orientation, in order to remove the intrinsic translational asymmetry in the B-factor values for this structure.

### 2.4 Structural analysis of CPD susceptibility

A custom python program was developed to use to calculate the average distance and torsion angle values between the C5-C6 double bonds of neighboring pyrimidine sequences, using our previously described method [22], [23]. To calculate distance, the program averaged the x, y, and z coordinates of each C5–C6 bond, then calculated the distance between the resulting midpoints of bonds of neighboring pyrimidine bases. To calculate the torsion angle of neighboring C5–C6 double bonds, the coordinates of the 5′ C5, 5′ C6, 3′ C6 and 3′ C5 were used to calculate the improper torsion (or dihedral) angle between the neighboring C5–C6 double bonds, as previously described [23]. The calculated distance and torsion angle values were then assigned to half integer positions between those of the parent nucleotides (e.g., distance and torsion angle values for pyrimidines at position +10 and +11 relative to the nucleosome dyad were assigned a position of +10.5). Distance and torsion angle averages were separately categorized as minor “in”, “out”, or “in-between” positions, depending on whether their positions were determined to be part of a minor groove that faces towards the histone octamer (minor-in), away from the histone octamer (minor-out), or in-between, respectively. These categories were adapted from previous studies [16]. Distance and torsion angle values at symmetric positions across the nucleosome dyad were combined and averaged (e.g., positions −10 and +10 from the dyad axis were combined and averaged). Comparison with CPD enrichment was performed using Pearson correlation analysis in Graphpad Prism software.

To determine the impact of distance and torsion angle independently on CPD enrichment, we divided the distance and torsion angle measurements for nucleosomal DNA into quartiles. Based on this analysis, distances of 4.17 Å or lower were designated ‘low’ (i.e., bottom quartile) and distances of 4.71 Å or higher were designated ‘high’ (i.e., top quartile). Similarly, torsion angles of 30.36 degrees or lower were likewise designated ‘low’ (i.e., bottom quartile) and torsion angles of 41.72 degrees or higher were designated ‘high’ (i.e., top quartile). From these, CPD enrichment values derived from five distinct categories were compared: low distances and low torsion angles, low distances and high torsion angles, intermediate distances and intermediate torsion angles (i.e., neither high nor low), high distances and low torsion angles, and high distances and high torsion angles. The CPD enrichment value corresponding to each position in a nucleosome structure that matched one of these structural categories was compiled for all nucleosome structures and analyzed by one-way ANOVA using Tukey’s multiple comparisons test.

### 2.5 Lomb-scargle periodicity analysis in nucleosomal DNA

The periodicities of B-factor, distances, torsion angles, and CPD enrichment in nucleosomes were determined using a Lomb-Scargle analysis. A custom R script was used to analyze the peak period, normalized power, signal-to-noise ratio, and significance of each separate dataset. Only the averaged values of each data type at each position within nucleosomal DNA were used for this analysis. Periods in the range between 5 bp and 25 bp were tested for each of these datasets. A second custom R script was then used to export all tested periodicities and their corresponding normalized powers to generate a periodogram using Graphpad Prism software.

## 3 Results

### 3.1 DNA mobility in nucleosome structures does not significantly correlate with CPD enrichment in nucleosomes

**Fig. 1:**
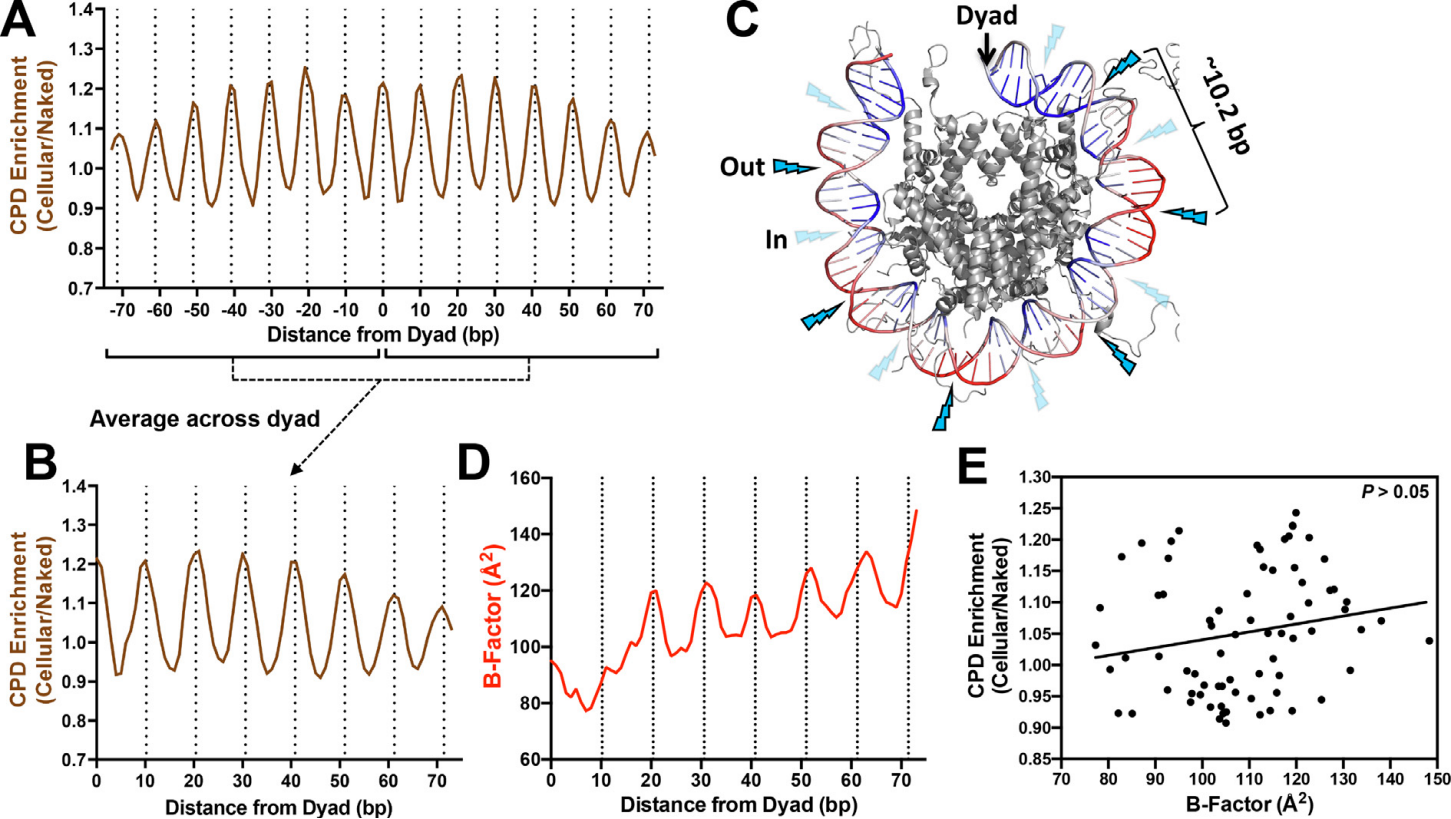
DNA mobility correlates poorly with CPD enrichment. (A) Analysis of CPD enrichment in ∼10,000 strongly positioned nucleosomes across the yeast genome [29]. CPD enrichment values are derived from CPD-seq data from UV-irradiated yeast cells normalized by CPD-seq data from UV-irradiated yeast genomic DNA (i.e., naked DNA) irradiated *in vitro*[9]. CPD enrichment is plotted relative to the position of the nucleotide from the central nucleosome dyad. Minor-out rotational settings are indicated with dashed lines. (B) CPD enrichment was combined for symmetric positions across the dyad (i.e., CPD data for positions −10 and +10 from the dyad were combined), resulting in a weighted average of CPD enrichment associated with different distances from the dyad. Minor-out rotational settings are indicated with dashed lines. (C) Structure of nucleosome (PDB ID: 1KX5) with B-factor values indicated by the coloring of the DNA molecule. Blue color indicates low B-factor and red color indicates relatively high B-factor. B-factor is generally lower at minor-in positions (indicated with faded lightning bolt) and higher at minor-out positions (indicated with bright lightning bolt). B-factor is also lower near the nucleosome dyad. Only half of the DNA molecule is shown for clarity. Image was generated using pymol. (D) B-factor (a measure of DNA mobility) is increased at minor-out positions in nucleosomes, but is generally decreased near the nucleosome dyad and elevated near the DNA ends. B-factor was computed from a compendium of ∼180 nucleosome structures and combined for symmetric positions across the nucleosome dyad (see above). (E) B-factor only weakly correlates with CPD enrichment in nucleosomal DNA (r = 0.193, *P* > 0.05 based on Pearson correlation analysis). The linear regression line is depicted. (For interpretation of the references to color in this figure legend, the reader is referred to the web version of this article.)

Previous analysis of published CPD-seq data from yeast [9] or human cells [10] indicates that CPD formation is significantly modulated in nucleosomes, with higher CPD formation at minor-out positions and lower CPD formation at minor-in positions. We confirmed these findings using our published CPD-seq data for UV-irradiated yeast cells [9]. We focused on yeast since it has arguably the highest-resolution nucleosome map, which is derived from a chemical cleavage method that precisely defines the location of the nucleosome dyad axis [29], and because of the plethora of high-resolution CPD damage mapping data available in yeast [9]. CPD formation in UV irradiated yeast cells was normalized to parallel CPD-seq experiments derived from isolated yeast genomic DNA that was UV-irradiated [9], to account for any potential sequence biases in the nucleosome DNA. The resulting CPD enrichment (i.e., CPDs in cellular relative to CPDs in naked DNA) revealed a clear periodicity in nucleosomes (period = 10.15 bp; see Supplementary Fig. S1), with peak CPD enrichment at minor-out positions (dashed lines in Fig. 1A, upper panel) and troughs of CPD enrichment at minor-in positions (Fig. 1A), consistent with previous results. Because CPD formation (and the nucleosome structure as a whole) is symmetric across the nucleosome dyad, we combined CPD levels at symmetric positions across the dyad (e.g., weighted average of CPD levels at positions −10 and +10 from the dyad, at positions −11 and +11, etc.) for all subsequent analysis (Fig. 1A,B).

To assess whether differences in DNA mobility is a potential cause of CPD modulation in nucleosomes, we analyzed atomic B-factor values from nucleosome structures. B-factor is a commonly-used measure of DNA or protein mobility in structures [31], and visualization of B-factor values in an example nucleosome structure indicates that B-factor is generally elevated at minor-out relative to minor-in positions (Fig. 1C), consistent with a previous report [14]. B-factor values for all DNA backbone atoms were averaged across a compendium of 181 high-resolution (<3.5 Å) nucleosome structures (Supplementary Fig. S2). Custom scripts were developed to calculate the average B-factor of the DNA backbone along the nucleosomal DNA backbone in each structure. This analysis revealed B-factor peaks near minor-out positions in nucleosomes, consistent with previous reports, which roughly correlated with peaks of CPD enrichment (compare Fig. 1B and D). While this pattern is apparent from positions ∼15 bp to ∼73 bp relative to the nucleosomal dyad, nucleosomal DNA immediately adjacent to the dyad axis (within ∼15 bp) lacks this periodicity and B-factor remained consistently low and no longer correlated with CPD enrichment (Fig. 1D). This heterogeneity in B-factor across the nucleosomal DNA resulted in a very weak rotational periodicity (∼10.5 bp) that was only marginally significant (*P* = 0.0338; Supplementary Fig. S3). For these mono-nucleosome structures, B-factor was generally lowest near the dyad axis and highest near the edge of nucleosomal DNA (Fig. 1D), as expected. However, CPD enrichment did not show this trend, as some of the highest peaks of CPD enrichment were at positions adjacent to the nucleosome dyad (i.e., positions 0 and 10 bp from the dyad axis; see Fig. 1B), where B-factor was generally low.

To more rigorously test the relationship between DNA mobility and CPD enrichment, we analyzed the correlation between average B-factor across the compendium of nucleosome structures and CPD enrichment at each position relative to nucleosome dyad. This analysis revealed that B-factor and CPD enrichment were poorly correlated in nucleosomes (r = 0.193; Fig. 1E), consistent with the observations described above. These findings indicate that B-factor is not significantly associated with CPD enrichment in nucleosomal DNA (*P* > 0.05), indicating that DNA mobility may not be the primary explanation for CPD modulation in nucleosomes. Similar results were obtained when we analyzed normalized B-factors (Supplementary Fig. S4), which only poorly correlated with CPD enrichment in nucleosomal DNA.

**Fig. 2:**
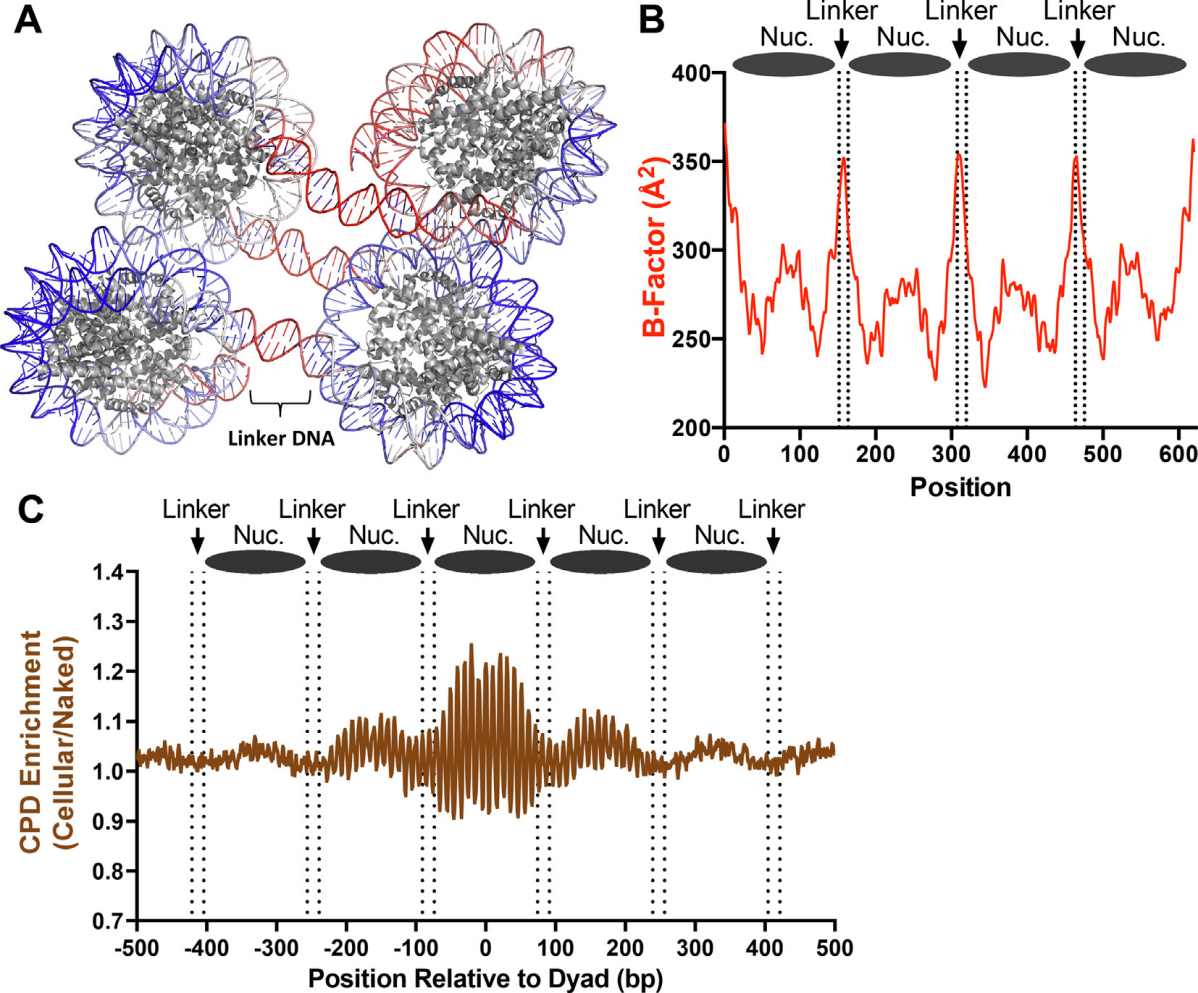
CPD enrichment correlates poorly with DNA mobility in linker DNA. (A) Visualization of DNA B-factor values in a structure of a tetranucleosome (PDB ID: 5OY7), with high B-factor colored red and low B-factor colored blue. Linker DNA between each nucleosome (∼12 bp) has a high B-factor, indicating linker DNA is significantly more mobile than nucleosomal DNA. Image was generated using pymol. (B) Plot of DNA backbone B-factor for tetranucleosome structure (PDB ID: 5OY7). Approximate locations of linker DNA in structure are highlighted with dashed lines. The two DNA strands are aligned in a 5′-3′ orientation and averaged to remove intrinsic B-factor translational asymmetry in the original structure. Note that B-factor is also somewhat elevated at dyad positions, potentially due to elevated mobility specific to the tetranucleosome conformation or reflecting alternative static conformations of the tetranucleosome present in the crystal. (C) Analysis of CPD enrichment in linker DNA immediately adjacent to yeast nucleosomes, derived from published yeast CPD-seq data [9]. Analysis was centered on dyad position of ∼10,000 strongly positioned nucleosomes in yeast [29]. Approximate locations of linker DNA is indicated with dashed lines. This analysis indicates CPD formation is not enriched in linker DNA in UV irradiated yeast. (For interpretation of the references to color in this figure legend, the reader is referred to the web version of this article.)

As a further test, we analyzed B-factor and CPD enrichment in linker DNA regions immediately adjacent to nucleosomes. Since linker regions are typically not bound by histone proteins when histone H1 is absent, these regions should have high DNA mobility, and therefore high CPD enrichment if the DNA mobility model is correct. For this purpose, we analyzed a structure of a chain of four nucleosomes (i.e., a tetranucleosome; see Fig. 2A) that lacked histone H1 and contained ∼11–12 bp of linker DNA between each nucleosome [32]. As expected, these linker DNA segments had higher B-factor than the adjacent nucleosomal DNA (Fig. 2B and Supplementary Fig. S5). However, analysis of CPD-seq data for linker regions immediately adjacent to strongly positioned nucleosomes in yeast indicated that CPD enrichment is slightly decreased in linker regions relative to the nucleosome core (Fig. 2C), even though yeast lacks a canonical histone H1 protein [33]. These findings are consistent with previous biochemical analysis of CPD formation in dinucleosomes indicating that CPD formation is not elevated in linker DNA [19]. In summary, this analysis suggests that even though linker regions have high mobility, they are not associated with elevated CPD enrichment in UV-irradiated cells.

### 3.2 Periodic alterations in nucleosomal DNA structure correlate with CPD enrichment

**Fig. 3:**
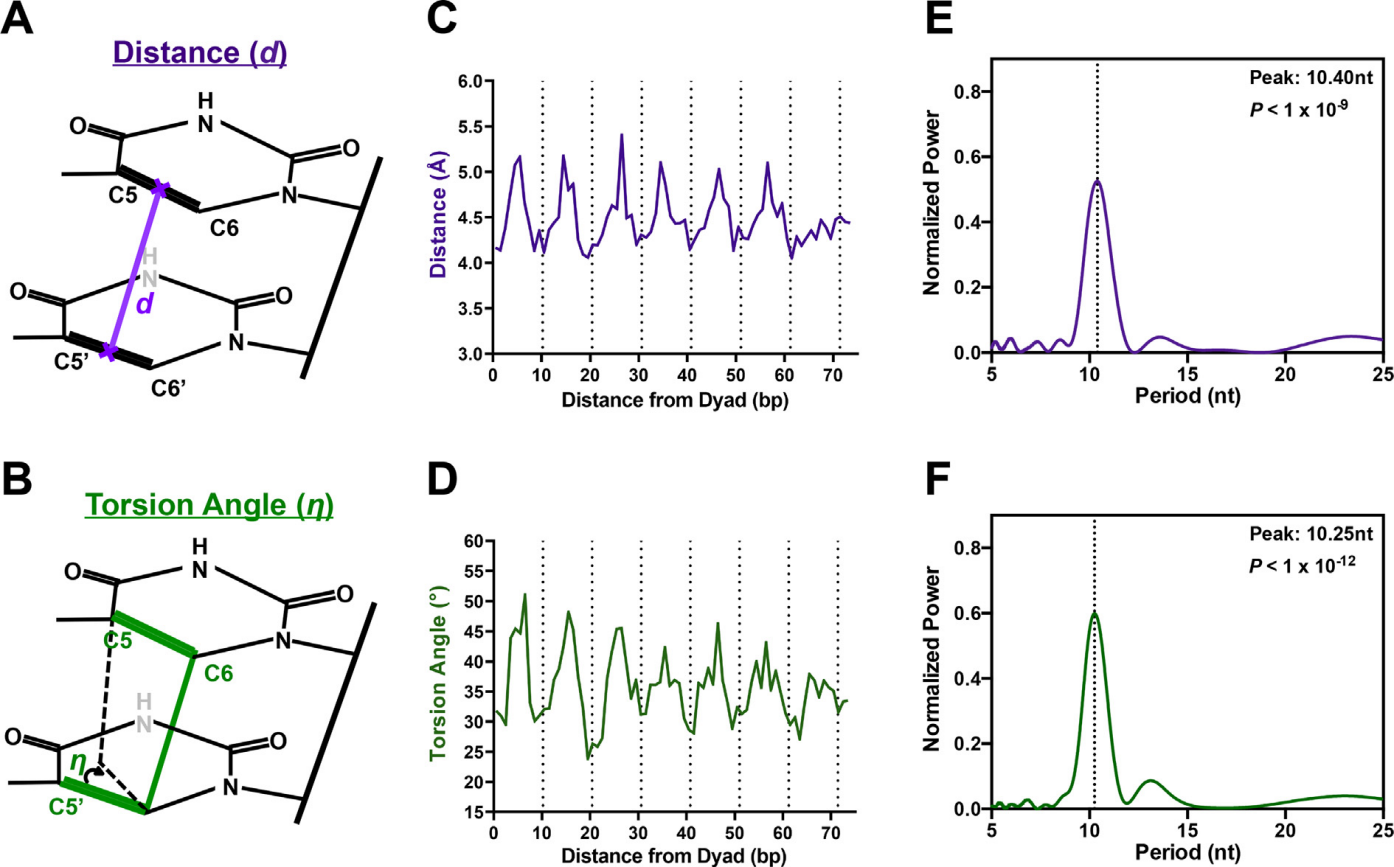
Periodic changes in the distance and torsion angle of C5–C6 double bonds of neighboring pyrimidines in nucleosome structures. (A) Schematic showing that the distance measurements were made between the midpoints of the C5–C6 double bonds of adjacent pyrimidines. (B) Schematic showing how the measurement of the improper torsion angle were made for the 5′ C5-C6 and 3′ C5-C6 bonds of adjacent pyrimidines. (C) Average C5-C6 distances between neighboring pyrimidines show periodic changes in nucleosomal DNA, with smaller distances at minor-out positions (dashed lines) and larger distances at minor-in positions. Distances were calculated from a compendium of ∼180 nucleosome structures. (D) Average torsion angle between C5-C6 double bonds of neighboring pyrimidines also show periodic changes in nucleosomal DNA, with smaller angles near minor-out positions and larger angles at minor-in positions. Torsion analges were calculated from a compendium of ∼180 nucleosome structures. (E-F) Lomb-Scargle analysis of periodicity of (E) average C5-C6 distance measurements and (F) average torsion angles revealed a significant peak periodicities of ∼10.4 and ∼10.3 bp, respectively.

Alternatively, it is possible that distortions in the DNA as it wraps around the histone octamer result in periodic (and static) DNA conformations that modulate CPD formation. We have recently shown that transcription factor binding-induced changes in the distance and relative torsion angle between the C5-C6 double bonds of neighboring pyrimidines (Fig. 3A) can predict their susceptibility to CPD formation [22], [23]. In general, smaller distance and torsion angle values appear to be associated with elevated CPD formation, while higher values result in diminished CPD formation [22], [23], consistent with previous biophysical studies [6], [7]. However, it is not known if these structural parameters are altered in nucleosomes.

To test this idea, we analyzed the distance and torsion angle between C5-C6 double bonds of neighboring pyrimidine sequences in our compendium of 181 high-resolution nucleosomes structures. This analysis revealed a clear periodicity in both the distance and torsion angles along the nucleosomal DNA (Fig. 3C,D). Both distance and torsion angle tended to have favorably low values at minor-out locations (dashed lines in Fig. 3C,D) and unfavorably high values at minor-in positions. This pattern was apparent both in the average distance and torsion angle values for all nucleosome DNA sequences (Fig. 3C,D), as well as in box plots of individual values for each nucleosome structure (Supplementary Fig. S6). Both the average distance and torsion angle exhibited a significant ∼10.4 bp and 10.25 bp periodicity, respectively (Fig. 3E,F), which roughly matched the observed CPD periodicity (Supplementary Fig. S1). Notably, distance and torsion angle showed stronger periodicity proximal to the nucleosome dyad than in distal regions (Supplementary Fig. S7), which could potentially explain why CPD enrichment has an apparent stronger periodicity near the nucleosome dyad (Figs. 1A,B and 2C).

**Fig. 4:**
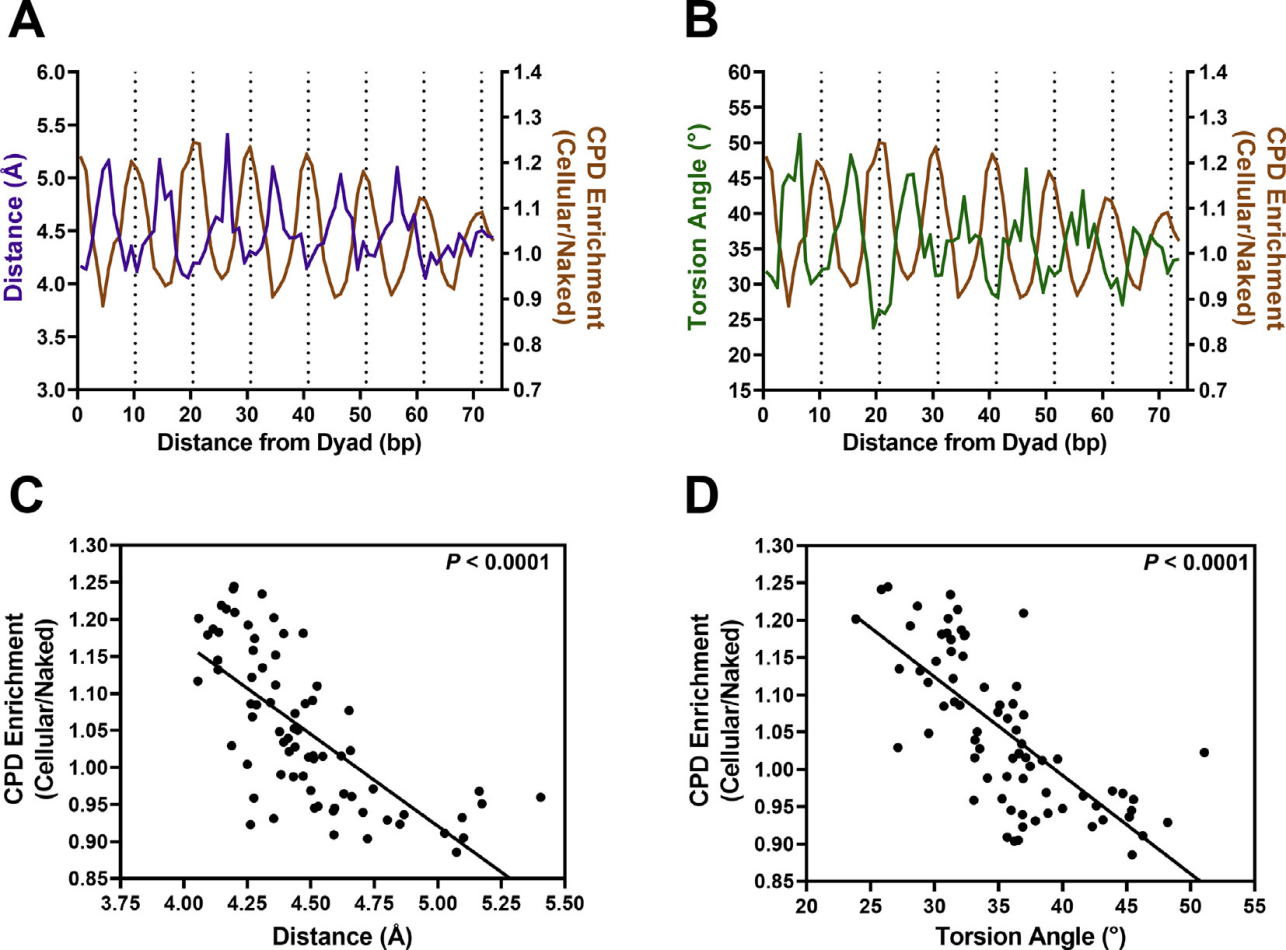
Distance and torsion angles of C5-C6 bonds between neighboring pyrimdines show a striking negative correlation with CPD enrichment in nucleosomal DNA. (A-B) Average C5-C6 distance and torsion angle are negatively correlated with CPD enrichment in nucleosomal DNA. Average distance and torsion angle values were calculated from the compendium of ∼180 nucleosomes structures (see Fig. 3). CPD enrichment data are derived from yeast CPD-seq data analyzed at ∼10,000 strongly positioned nucleosomes (see Fig. 1). (C,D) Average distance and torsion angle between C5-C6 double bonds of neighboring pyrimidines show a highly significant negative correlation with CPD enrichment in nucleosome DNA, based on Pearson correlation analysis (r = −0.703 and −0.727, respectively; *P* < 0.0001). The linear regression line is also depicted.

Comparison of these structural features with CPD enrichment revealed that average distance and torsion angle of the C5-C6 bonds of adjacent pyrimidine bases were negatively correlated with CPD formation (Fig. 4A,B). Average distance and torsion angle tended to be lowest at minor-out positions, where CPD enrichment is highest, and tended to be highest at minor-in positions, where CPD enrichment is lowest (Fig. 4A,B). To more rigorously test these associations, we analyzed the correlation between average distance or torsion angle and CPD enrichment at each position relative to nucleosome dyad. This analysis revealed that distance and torsion angle of neighboring C5-C6 bonds were both negatively correlated with CPD enrichment (r = −0.703 and −0.727, respectively; Fig. 4C,D); these negative correlations were highly significant (*P* < 0.0001). These findings indicate that lower distance and torsion angles at minor-out positions may explain elevated CPD formation at these same DNA positions, while increased distance and torsion angles at minor-in positions may result in suppressed CPD formation at these locations.

**Fig. 5:**
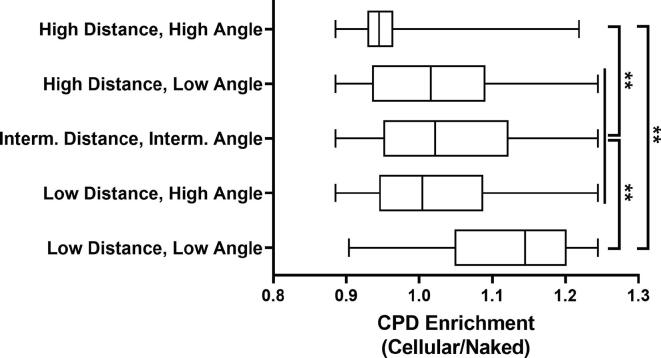
Coordinate changes distance and torsion angle between neighboring pyrimidines modulate CPD formation. Box and whisker plot of CPD enrichment values associated with nucleosomal DNA positions with high (top quartile) or low (bottom quartile) of distance or torsion angle values, based on a compendium of ∼180 nucleosome structures. Each nucleosome structure (and DNA chain) was analyzed independently. Intermediate values were in neither the top or bottom quartile. Significance was determined by one-way ANOVA using Tukey’s multiple comparisons test. Note, the high distance, low torsion angle category was also significantly different than the intermediate distance, intermediate torsion angle category.

To determine whether the distance or torsion angle of neighboring C5-C6 bonds had a greater impact on CPD enrichment, we categorized dipyrimidine base steps as low (bottom quartile) or high (top quartile) for each structural category across all 181 nucleosome structures (see Experimental Procedures). This analysis indicated that nucleosomal DNA positions with intermediate distances and intermediate torsion angles (i.e., neither high nor low) had an average CPD enrichment score of nearly 1, as expected. In contrast, nucleosome positions where both distance and torsion angle were low had significantly higher CPD enrichment (i.e., CPD enrichment >1; Fig. 5). Similarly, if distance and torsion angle were both high at a nucleosome position, this was generally associated with decreased CPD enrichment (Fig. 5). Notably, nucleosomal DNA positions where one structural category was high and the other low had CPD enrichment values that were roughly similar to that of the intermediate distance and torsion angle category (Fig. 5). This analysis suggests that both distance and torsion angle of C5-C6 bonds in neighboring bases affect CPD formation in nucleosomes, and that high CPD formation tended to be observed only if both parameters were coordinately low, and low CPD formation was observed if both parameters were coordinately high.

## 4 Discussion

While previous studies have indicated that UV-induced CPD formation is significantly modulated in nucleosomal DNA, the molecular mechanism responsible for this modulation was previously unclear. Here, we report the analysis of ∼180 published nucleosome structures, which revealed that periodic changes in the conformation of nucleosomal DNA as it bends around the histone octamer modulates its susceptibility to UV damage. Our analysis indicates that the distance and torsion angle between the CPD-forming C5-C6 double bonds of neighboring pyrimidines show a striking periodic trend in nucleosomal DNA, with favorable distance and torsion angle values at minor-out rotational settings, and generally unfavorable values at minor-in positions. Hence, these structural parameters strongly correlate with, and potentially account for, CPD enrichment in nucleosomes from UV-irradiated cells.

While DNA mobility, as measured by atomic B-factor, also exhibits a periodic trend in nucleosomal DNA, these periodic variations in DNA mobility were poorly correlated with CPD formation. This discrepancy is particularly apparent near the nucleosome dyad and in adjacent linker DNA, where differences in DNA mobility do not translate to changes in CPD formation. Although elevated DNA mobility at minor-out positions and reduced mobility at minor-in positions may also affect CPD formation, our analysis indicates that differences in DNA mobility is not the primary cause of CPD modulation in nucleosomes.

Taken together, these findings indicate that the sharp bending of the DNA around the histone octamer results in DNA conformations that modulate UV damage susceptibility. This sharp bending is likely achieved by altering the roll and slide parameters of individual base steps that result in the nucleosomal DNA bending into the major groove at minor-out positions and into the minor groove at minor-in positions [14], [15]. We propose that bending into the minor groove at minor-in positions, which results in DNA overwinding due to increased helical twist [15], is likely responsible for the unfavorably large distance and torsion angles between the C5-C6 double bonds of neighboring pyrimidines. In contrast, bending into the major groove, which results in DNA underwinding due to reduced helical twist [15], may underlie the smaller (and more favorable) distance and torsion angles found at minor-out positions in nucleosome stuctures. Hence, the requirement for sharp DNA bending around the histone octamer is likely responsible for increased CPD formation at minor-out positions. This is consistent with genome-wide CPD-seq data [9], [10] indicating that CPD formation at minor-out positions in nucleosomes is elevated relative to unbound naked DNA or flexible linker regions. This model can also potentially explain why somatic mutation rates in human skin cancers are specifically elevated at minor-out positions in nucleosomes [11], [23], [30]. This DNA bending hypothesis is consistent with ideas proposed >30 years ago [19], [20], prior to the publication of the first high-resolution nucleosome structure. Notably, these same structural parameters can also explain the modulation of CPD formation at transcription factor binding sites [22], [23], indicating that a common molecular mechanism may explain patterns of UV damage and mutagenesis associated with nucleosomes and other DNA-bound proteins.

## CRediT authorship contribution statement

**Bastian Stark:** Conceptualization, Data curation, Formal analysis, Investigation, Methodology, Software, Validation, Writing – original draft. **Gregory M.K. Poon:** Conceptualization, Funding acquisition, Project administration, Formal analysis, Validation, Writing – review & editing. **John J. Wyrick:** Conceptualization, Funding acquisition, Project administration, Formal analysis, Methodology, Software, Validation, Writing – review & editing.

## Declaration of Competing Interest

The authors declare that they have no known competing financial interests or personal relationships that could have appeared to influence the work reported in this paper.
