# Supplementary material for: Molecular mechanism of UV damage modulation in nucleosomes
Source: Comput Struct Biotechnol J. 2022 Sep 14;20:5393–400. doi: 10.1016/j.csbj.2022.08.071 (PMC9529667; doi:10.1016/j.csbj.2022.08.071)
Supplement: Supplementary data 1 [file mmc1.pdf]

Supplementary Materials

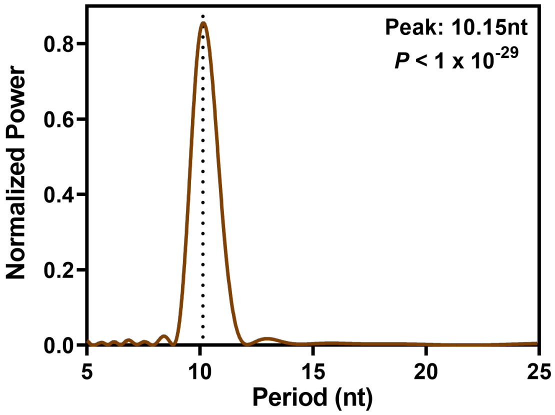

Supplementary Fig. S1: Lomb-Scargle analysis of CPD enrichment in strongly positioned nucleosomes in yeast reveals a significant peak periodicity of 10.15 bp. The brown line indicates the measured normalized power of given tested periodicities between 5 and 25 bp.

181 Structures:

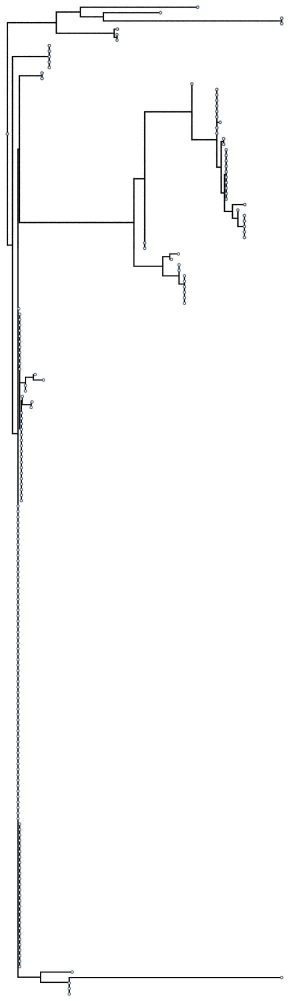

Supplementary Fig. S2: Cladogram of the nucleosome sequences used for this analysis. Branch lengths correspond to measure of sequence dissimilarity. The two major groups into which most structures fell were alpha satellite DNA and 601 sequence, though a large number of other sequences were present as well. Tree was generated with ClustalW (https://www.genome.jp/tools-bin/clustalw) using FastTree visualization [34].

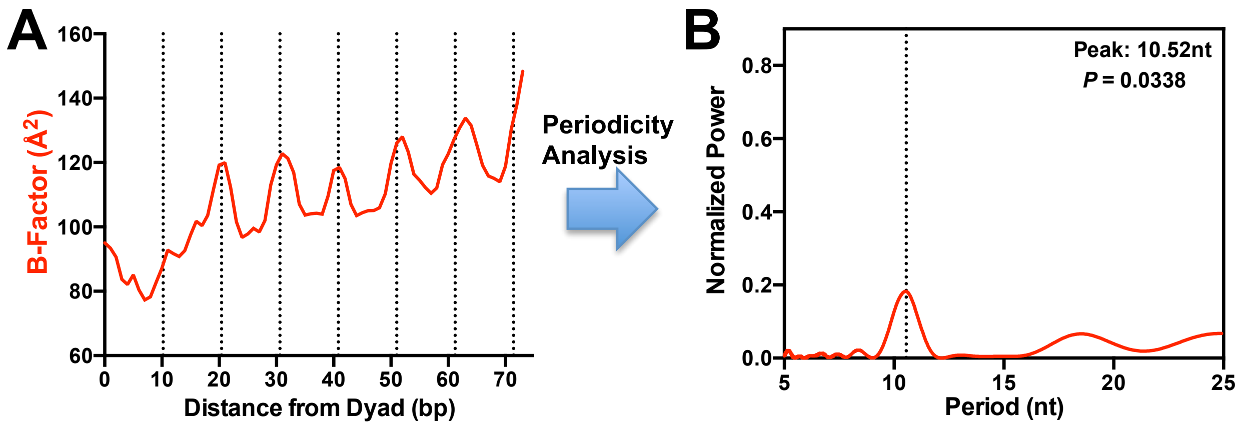

Supplementary Fig. S3: (A,B) Analysis of B-factor periodicity in nucleosome structures.

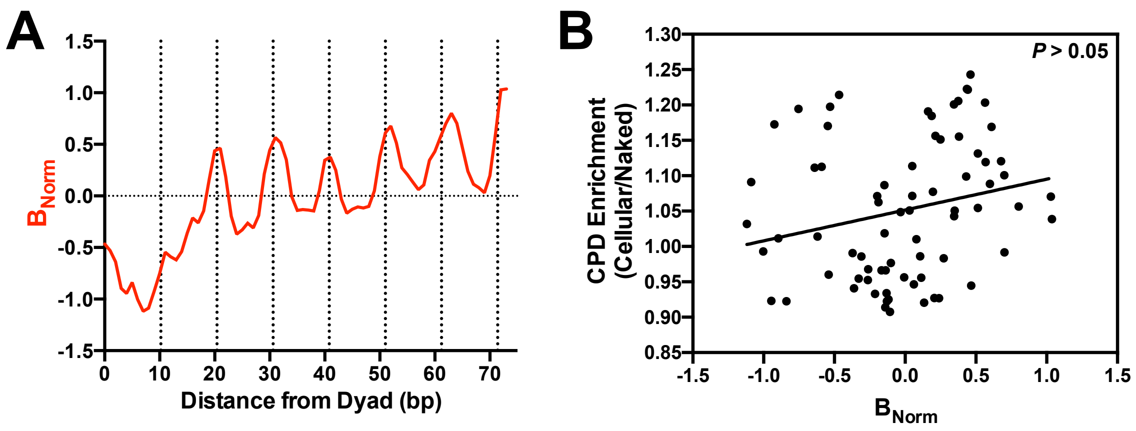

Supplementary Fig. S4: (A) Normalized B-factor (B_{Norm}, a measure of DNA mobility) shows a similar pattern in nucleosomes as B-factor. (B) Normalized B-factor only weakly correlates with CPD enrichment in nucleosomal DNA (r = 0.2245, $P > 0.05$ based on Pearson correlation analysis). The linear regression line is depicted.

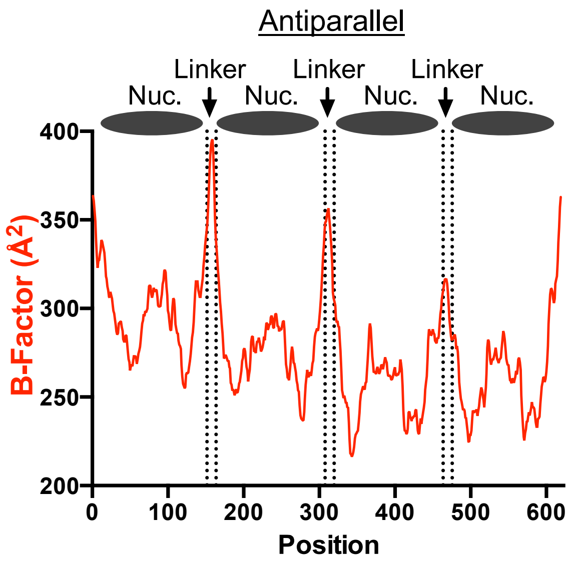

Supplementary Fig. S5: Plot of DNA backbone B-factor for the tetranucleosome structure (PDB ID: 5OY7). Approximate locations of linker DNA in structure are highlighted with dashed lines. The two DNA strands were averaged and plotted in the antiparallel orientation.

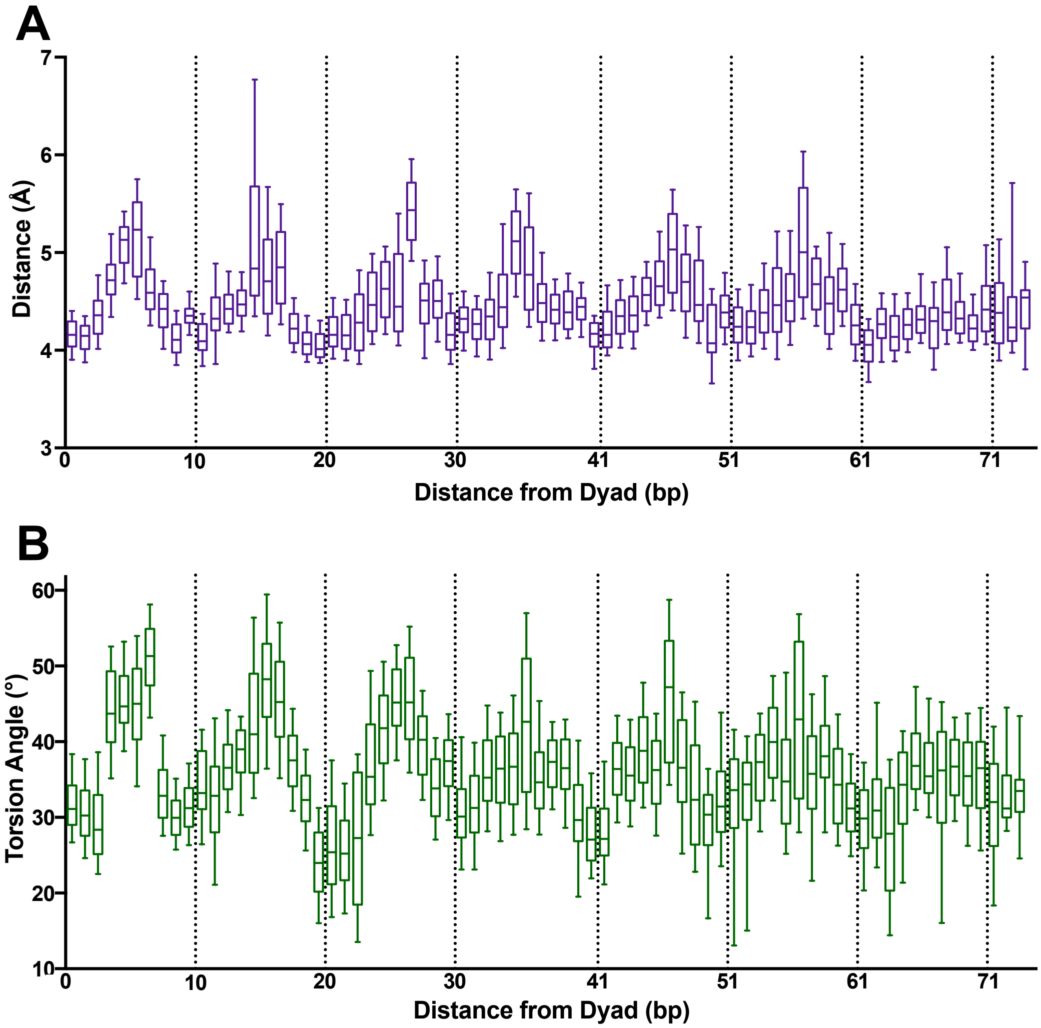

Supplementary Fig. S6: Box and whisker plot of C5-C6 bond (A) distance and (B) torsion angle of neighboring pyrimidines compiled from a compendium of ~180 nucleosome structures. Whiskers indicate range of 10th-90th percentile values. Both distance and torsion angle followed a consistent periodic pattern of low values at minor-out positions and higher values at minor-in positions.

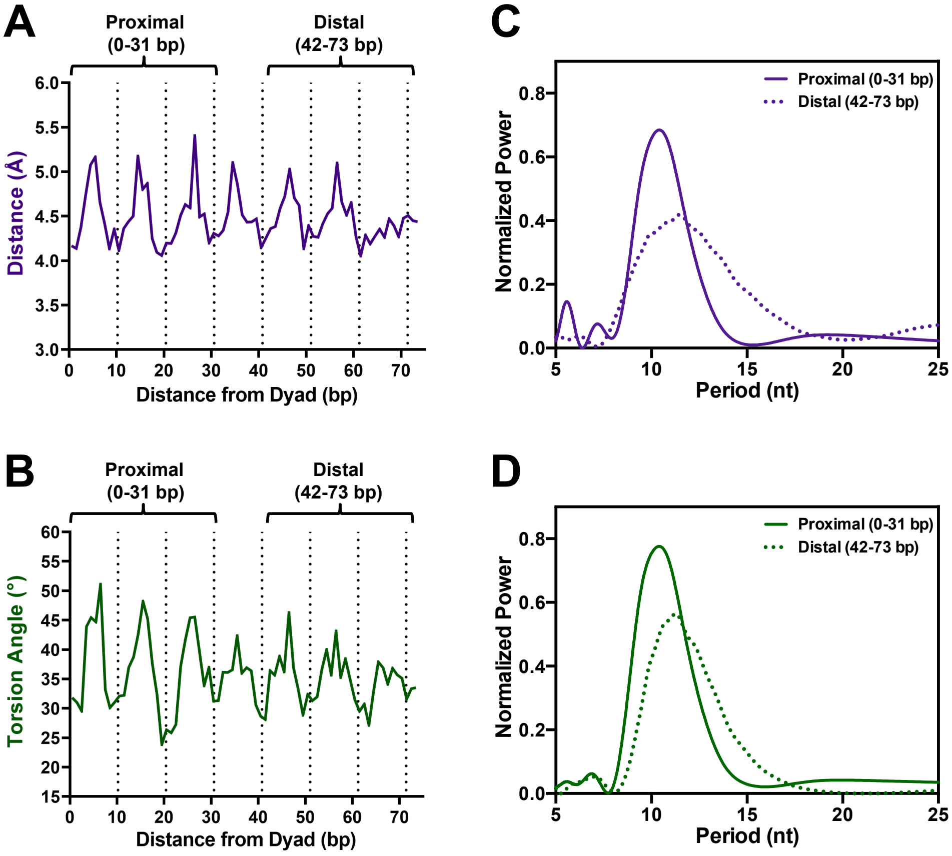

Supplementary Fig. S7: (A,B) Average C5-C6 distances and torsion angle between neighboring pyrimidines show periodic changes in nucleosomal DNA. Same as Fig. 3C,D. Dyad proximal and distal regions are indicated. (C,D) Lomb-Scargle analysis of periodicity of (C) average C5-C6 distance measurements and (D) average torsion angles for dyad proximal and distal regions are depicted.

Supplementary References

34. Price, M. N., Dehal, P. S., and Arkin, A. P. (2010) FastTree 2--approximately maximum-likelihood trees for large alignments. *PloS one* **5**, e9490
