# Supplementary material for: Molecular mechanism of UV damage modulation in nucleosomes
Source: Comput Struct Biotechnol J. 2022 Sep 14;20:5393–400. doi: 10.1016/j.csbj.2022.08.071 (PMC9529667; doi:10.1016/j.csbj.2022.08.071)
Supplement: Supplementary data 2 [file mmc2.docx]

**Supplementary Table S1:** PDB IDs of nucleosome structures used in the analysis.

| PDB ID | Chain ID: | Dyad Position |
| --- | --- | --- |
| 1aoi | I | 73 |
| 1aoi | J | 220 |
| 1eqz | I | 73 |
| 1eqz | J | 220 |
| 1f66 | I | 73 |
| 1f66 | J | 220 |
| 1id3 | I | 73 |
| 1id3 | J | 220 |
| 1kx3 | I | 0 |
| 1kx3 | J | 0 |
| 1kx4 | I | 0 |
| 1kx4 | J | 0 |
| 1kx5 | I | 0 |
| 1kx5 | J | 0 |
| 1m1a | I | 73 |
| 1m1a | J | 220 |
| 1m18 | I | 73 |
| 1m18 | J | 220 |
| 1m19 | I | 73 |
| 1m19 | J | 220 |
| 1p3a | I | 73 |
| 1p3a | J | 220 |
| 1p3b | I | 73 |
| 1p3b | J | 220 |
| 1p3f | I | 73 |
| 1p3f | J | 220 |
| 1p3g | I | 73 |
| 1p3g | J | 220 |
| 1p3i | I | 73 |
| 1p3i | J | 220 |
| 1p3k | I | 73 |
| 1p3k | J | 220 |
| 1p3l | I | 73 |
| 1p3l | J | 220 |
| 1p3m | I | 73 |
| 1p3m | J | 220 |
| 1p3o | I | 73 |
| 1p3o | J | 220 |
| 1p3p | I | 73 |
| 1p3p | J | 220 |
| 1p34 | I | 73 |
| 1p34 | J | 220 |
| 1s32 | I | 73 |
| 1s32 | J | 220 |
| 1u35 | I | 73 |
| 1u35 | J | 218 |
| 1zla | I | 73 |
| 1zla | J | 220 |
| 2cv5 | I | 73 |
| 2cv5 | J | 220 |
| 2f8n | I | 73 |
| 2f8n | J | 218 |
| 2nqb | I | 73 |
| 2nqb | J | 220 |
| 2nzd | I | 0 |
| 2nzd | J | 0 |
| 2pyo | I | 0 |
| 2pyo | J | 0 |
| 3a6n | I | 73 |
| 3a6n | J | 220 |
| 3afa | I | 73 |
| 3afa | J | 220 |
| 3av1 | I | 73 |
| 3av1 | J | 220 |
| 3av2 | I | 73 |
| 3av2 | J | 220 |
| 3ayw | I | 73 |
| 3ayw | J | 220 |
| 3aze | I | 73 |
| 3aze | J | 220 |
| 3azf | I | 73 |
| 3azf | J | 220 |
| 3azg | I | 73 |
| 3azg | J | 220 |
| 3azh | I | 73 |
| 3azh | J | 220 |
| 3azi | I | 73 |
| 3azi | J | 220 |
| 3azj | I | 73 |
| 3azj | J | 220 |
| 3azk | I | 73 |
| 3azk | J | 220 |
| 3azl | I | 73 |
| 3azl | J | 220 |
| 3azm | I | 73 |
| 3azm | J | 220 |
| 3azn | I | 73 |
| 3azn | J | 220 |
| 3b6f | I | 0 |
| 3b6f | J | 0 |
| 3b6g | I | 0 |
| 3b6g | J | 0 |
| 3c1c | I | 73 |
| 3c1c | J | 220 |
| 3kuy | I | 0 |
| 3kuy | J | 0 |
| 3kwq | I | 73 |
| 3kwq | J | 220 |
| 3kxb | I | 73 |
| 3kxb | J | 220 |
| 3lel | I | 0 |
| 3lel | S | 0 |
| 3lel | J | 0 |
| 3lel | T | 0 |
| 3lja | I | 0 |
| 3lja | J | 0 |
| 3lz0 | I | 0 |
| 3lz0 | J | 0 |
| 3lz1 | I | 0 |
| 3lz1 | J | 0 |
| 3mgp | I | -1 |
| 3mgp | J | 1 |
| 3mgq | I | -1 |
| 3mgq | J | 1 |
| 3mgr | I | 0 |
| 3mgr | J | 0 |
| 3mnn | I | 0 |
| 3mnn | J | 0 |
| 3mvd | I | 74 |
| 3mvd | J | 74 |
| 3o62 | I | 73 |
| 3o62 | J | 220 |
| 3reh | I | 0 |
| 3reh | J | 0 |
| 3rei | I | 0 |
| 3rei | J | 0 |
| 3rej | I | 0 |
| 3rej | J | 0 |
| 3rek | I | 0 |
| 3rek | J | 0 |
| 3rel | I | 0 |
| 3rel | J | 0 |
| 3tu4 | I | 74 |
| 3tu4 | J | 74 |
| 3ut9 | I | 0 |
| 3ut9 | J | 0 |
| 3uta | I | 0 |
| 3uta | J | 0 |
| 3utb | I | 0 |
| 3utb | J | 0 |
| 3w96 | I | 73 |
| 3w96 | J | 220 |
| 3w97 | I | 73 |
| 3w97 | J | 220 |
| 3w98 | I | 73 |
| 3w98 | J | 220 |
| 3w99 | I | 73 |
| 3w99 | J | 220 |
| 3wa9 | I | 73 |
| 3wa9 | J | 220 |
| 3waa | I | 73 |
| 3waa | J | 220 |
| 3wkj | I | 73 |
| 3wkj | J | 220 |
| 3wtp | I | 73 |
| 3wtp | J | 220 |
| 3x1s | I | 73 |
| 3x1s | J | 220 |
| 3x1t | I | 73 |
| 3x1t | J | 220 |
| 3x1u | I | 73 |
| 3x1u | J | 220 |
| 3x1v | I | 73 |
| 3x1v | J | 220 |
| 4jjn | I | 74 |
| 4jjn | J | 74 |
| 4kud | I | 73 |
| 4kud | J | 220 |
| 4wu9 | I | 0 |
| 4wu9 | J | 0 |
| 4xzq | I | 74 |
| 4xzq | J | 221 |
| 4ys3 | I | 74 |
| 4ys3 | J | 221 |
| 4z5t | I | 73 |
| 4z5t | J | 220 |
| 4z66 | I | 74 |
| 4z66 | J | 221 |
| 5av5 | I | 0 |
| 5av5 | J | 0 |
| 5av6 | I | 0 |
| 5av6 | J | 0 |
| 5av8 | I | 0 |
| 5av8 | J | 0 |
| 5av9 | I | 0 |
| 5av9 | J | 0 |
| 5avb | I | 0 |
| 5avb | J | 0 |
| 5avc | I | 0 |
| 5avc | J | 0 |
| 5ay8 | I | 73 |
| 5ay8 | J | 220 |
| 5b0y | I | 73 |
| 5b0y | J | 220 |
| 5b0z | I | 73 |
| 5b0z | J | 220 |
| 5b1l | I | 73 |
| 5b1l | J | 220 |
| 5b1m | I | 73 |
| 5b1m | J | 220 |
| 5b2i | I | 0 |
| 5b2i | J | 0 |
| 5b2j | I | 0 |
| 5b2j | J | 0 |
| 5b31 | I | 73 |
| 5b31 | J | 220 |
| 5b32 | I | 73 |
| 5b32 | J | 220 |
| 5b33 | I | 73 |
| 5b33 | J | 220 |
| 5b40 | I | 73 |
| 5b40 | J | 220 |
| 5cpi | I | 73 |
| 5cpi | J | 74 |
| 5cpj | I | 73 |
| 5cpj | J | 74 |
| 5cpk | I | 73 |
| 5cpk | J | 73 |
| 5e5a | I | 73 |
| 5e5a | J | 220 |
| 5gsu | I | 73 |
| 5gsu | J | 220 |
| 5gt0 | I | 73 |
| 5gt0 | J | 220 |
| 5gt3 | I | 73 |
| 5gt3 | J | 220 |
| 5gtc | I | 73 |
| 5gtc | J | 220 |
| 5gxq | I | 73 |
| 5gxq | J | 220 |
| 5jrg | I | 73 |
| 5jrg | J | 73 |
| 5omx | I | 0 |
| 5omx | J | 0 |
| 5ong | I | 0 |
| 5ong | J | 0 |
| 5onw | I | 0 |
| 5onw | J | 0 |
| 5x7x | I | 73 |
| 5x7x | J | 220 |
| 5xf3 | I | 0 |
| 5xf3 | J | 0 |
| 5xf4 | I | 0 |
| 5xf4 | J | 0 |
| 5xf5 | I | 0 |
| 5xf5 | J | 0 |
| 5xf6 | I | 0 |
| 5xf6 | J | 0 |
| 5xm0 | I | 73 |
| 5xm0 | J | 220 |
| 5xm1 | I | 73 |
| 5xm1 | J | 220 |
| 5y0c | I | 73 |
| 5y0c | J | 220 |
| 5y0d | I | 73 |
| 5y0d | J | 220 |
| 5z23 | I | 73 |
| 5z23 | J | 220 |
| 5z30 | I | 73 |
| 5z30 | J | 220 |
| 5zbx | I | 73 |
| 5zbx | J | 220 |
| 6dzt | I | 74 |
| 6dzt | J | 74 |
| 6e0c | I | 74 |
| 6e0c | J | 74 |
| 6e0p | I | 73 |
| 6e0p | J | 73 |
| 6ipu | I | 0 |
| 6ipu | J | 0 |
| 6iq4 | I | 0 |
| 6iq4 | J | 0 |
| 6iro | I | 74 |
| 6iro | J | 74 |
| 6jou | I | 73 |
| 6jou | J | 220 |
| 6jr0 | I | 73 |
| 6jr0 | J | 220 |
| 6jr1 | I | 73 |
| 6jr1 | J | 220 |
| 6jxd | I | 0 |
| 6jxd | J | 0 |
| 6jyl | I | 74 |
| 6jyl | J | 74 |
| 6k1i | I | 0 |
| 6k1i | J | 0 |
| 6k1j | I | 0 |
| 6k1j | J | 0 |
| 6k1k | I | 0 |
| 6k1k | J | 0 |
| 6ke9 | I | 0 |
| 6ke9 | J | 0 |
| 6kvd | I | 73 |
| 6kvd | J | 220 |
| 6l9h | I | 0 |
| 6l9h | J | 0 |
| 6le9 | I | 0 |
| 6le9 | J | 0 |
| 6ler | S | 0 |
| 6ler | T | 0 |
| 6ler | I | 0 |
| 6ler | J | 0 |
| 6m4g | I | 74 |
| 6m4g | J | 74 |
| 6mup | I | 0 |
| 6mup | J | 0 |
| 6o1d | I | 73 |
| 6o1d | J | 73 |
| 6se6 | I | 0 |
| 6se6 | J | 0 |
| 6seg | I | 0 |
| 6seg | J | 0 |
| 6t79 | I | 72 |
| 6t79 | J | 74 |
| 6t93 | I | 74 |
| 6t93 | J | 74 |
| 6uph | I | 0 |
| 6uph | J | 0 |
| 6v2k | I | 73 |
| 6v2k | J | 220 |
| 6y5e | I | 77 |
| 6y5e | J | 77 |
| 6zhx | I | 0 |
| 6zhx | J | 0 |
| 6zhy | I | 0 |
| 6zhy | J | 0 |
| 7ea5 | I | 74 |
| 7ea5 | J | 74 |
| 7ea8 | I | 74 |
| 7ea8 | J | 74 |
| 7jo9 | I | 0 |
| 7jo9 | J | 0 |
| 7joa | I | 0 |
| 7joa | J | 0 |
| 7k5x | I | 99 |
| 7k5x | J | 99 |
| 7k5y | I | 99 |
| 7k5y | J | 99 |
| 7k60 | I | 99 |
| 7k60 | J | 99 |
| 7k61 | I | 99 |
| 7k61 | J | 99 |
| 7k63 | I | 99 |
| 7k63 | J | 99 |
| 7k78 | I | 73 |
| 7k78 | J | 220 |
| 7kbd | I | 74 |
| 7kbd | J | 74 |
| 7kbe | I | 74 |
| 7kbe | J | 74 |
| 7ktq | I | 84 |
| 7ktq | J | 84 |
| 7lya | I | 0 |
| 7lya | J | 0 |
| 7oh9 | I | 0 |
| 7oh9 | J | 0 |
| 7oha | I | 0 |
| 7oha | J | 0 |
| 7ohb | I | 0 |
| 7ohb | J | 0 |
| 7ohc | I | 0 |
| 7ohc | J | 0 |
| 7on1 | I | 0 |
| 7on1 | J | 0 |
| 7vbm | I | 0 |
| 7vbm | J | 0 |
